# Cardiac foetal reprogramming: a tool to exploit novel treatment targets for the failing heart

**DOI:** 10.1111/joim.13094

**Published:** 2020-06-17

**Authors:** A. van der Pol, M. F. Hoes, R. A. de Boer, P. van der Meer

**Affiliations:** ^1^ From the Department of Cardiology University Medical Center Groningen University of Groningen Groningen the Netherlands; ^2^ Perioperative Inflammation and Infection Group Department of Medicine Faculty of Medicine and Health Sciences University of Oldenburg Oldenburg Germany

**Keywords:** foetal gene programme, heart failure

## Abstract

As the heart matures during embryogenesis from its foetal stages, several structural and functional modifications take place to form the adult heart. This process of maturation is in large part due to an increased volume and work load of the heart to maintain proper circulation throughout the growing body. In recent years, it has been observed that these changes are reversed to some extent as a result of cardiac disease. The process by which this occurs has been characterized as cardiac foetal reprogramming and is defined as the suppression of adult and re‐activation of a foetal genes profile in the diseased myocardium. The reasons as to why this process occurs in the diseased myocardium are unknown; however, it has been suggested to be an adaptive process to counteract deleterious events taking place during cardiac remodelling. Although still in its infancy, several studies have demonstrated that targeting foetal reprogramming in heart failure can lead to substantial improvement in cardiac functionality. This is highlighted by a recent study which found that by modulating the expression of 5‐oxoprolinase (OPLAH, a novel cardiac foetal gene), cardiac function can be significantly improved in mice exposed to cardiac injury. Additionally, the utilization of angiotensin receptor neprilysin inhibitors (ARNI) has demonstrated clear benefits, providing important clinical proof that drugs that increase natriuretic peptide levels (part of the foetal gene programme) indeed improve heart failure outcomes. In this review, we will highlight the most important aspects of cardiac foetal reprogramming and will discuss whether this process is a cause or consequence of heart failure. Based on this, we will also explain how a deeper understanding of this process may result in the development of novel therapeutic strategies in heart failure.

## Graphical abstract

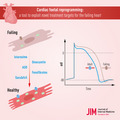

Content List ‐ Read more articles from the symposium: “Cardiovascular program and cardiovascular retreat”.

## Introduction

The mammalian heart is the first organ to develop during embryogenesis. As the foetal heart develops, several structural and functional modifications take place to form the four‐chambered adult heart. This process of maturation is in large part a result of increased volume and work load of the heart to maintain proper circulation throughout the growing body [1, 2, 3, 4]. Over the past decade, multiple advances in myocardial cell homeostasis and stem cell biology have enhanced our understanding of cardiac cellular differentiation and maturation. These findings coupled to our knowledge of heart failure (HF) have led to the discovery that cardiac injury in the adult heart leads to a switch in gene expression which to some extent resembles the expression pattern observed in the developing heart, a process known as ‘cardiac foetal reprogramming’. The exact reasons and mechanisms as to why the adult heart reverts back to a foetal‐like expression pattern remain unknown. However, it has been suggested that this process is an adaptive response to cope with adverse remodelling in the heart. Strikingly, it is still uncertain whether the re‐expression of foetal genes is an adaptive response that protects the heart during HF, or a maladaptive response that compounds the insult to an already weakened heart. In the present review, we summarize the current knowledge of the cardiac foetal gene programme, by looking at the expression profiles during cardiac development and disease, with a particular focus on cardiac metabolism, contractile machinery, electrophysiology and neurohormonal expression. Finally, we will explore how a better understanding of this process may lead to novel pathophysiological pathways and treatment targets to improve HF patient outcome.

## Foetal reprogramming in cardiac metabolism

Each contraction of the heart requires relatively large amounts of ATP [5]. With very low energy stores and a high ATP turnover, the metabolic activity of the heart is the highest of all organs in the body [6]. To meet energetic needs, the mature myocardium of the adult heart primarily utilizes fatty acids. Under certain conditions, the heart can also use pyruvate, lactate, acetate, amino acids, ketone bodies and phospho‐creatine [7]. Each of these substrates can be metabolized to generate acetyl coenzyme A (acetyl‐CoA), which in turn is essential for the production of substrates used in the oxidative phosphorylation pathway.

### From Glycolysis (foetal) to fatty acid oxidation (adult)

Foetal development occurs in a relative hypoxic environment [8]; therefore, ATP is ultimately generated through anaerobic glycolysis during foetal development. This hypoxic state results in high levels of HIF‐1α protein, which induces the transcription of major glycolysis key factors like glucose transporter (GLUT)‐1 and GLUT‐4 [9, 10, 11], hexokinase (HK)‐1 [9], lactate dehydrogenase (LDH)‐A [9, 10] and pyruvate dehydrogenase kinase (PDK)‐1 and PDK‐2 [12, 13]. In the foetal heart, GLUT‐1 is the major transporter of extracellular glucose, which is intracellularly converted to glucose‐6‐phosphate by HK‐1 [14, 15]. Furthermore, high expression levels of LDH‐A greatly contributes to the conversion of glycolysis‐derived pyruvate to lactate, consequently regenerating nicotinamide adenine dinucleotide (NAD^+^) from its reduced form (NADH), which is needed to sustain glycolysis [16]. Additionally, the foetal heart can also utilize lactate as main energy source [17, 18]. Combined, any additional lactate produced by LDH‐A can be efficiently oxidized in order to produce pyruvate and restore the levels of NADH required for continued ATP production through glycolysis.

After birth, cardiac metabolism does not switch to different substrates until 7 days postpartum, in lambs [18, 19]. In rabbits, it has been observed that circulating lactate levels fall 5‐7 mM to 0.5 mM in the first 2 hours after birth [20]. As such, lactate oxidation contributes notably less to ATP production. Moreover, glycolytic rates decrease from 44% to only 10% by day 7 after birth, in rabbits [21]. In concert with reduced glycolytic rates, fatty acid oxidation rates gradually increase towards levels observed in the hearts of adult animals [22]. Metabolic contribution by the various ATP‐generating pathways stabilizes around 3 weeks after birth with fatty acid oxidation as the main metabolic pathway, contributing to 89% of total ATP production [23, 24].

**Fig. 1:**
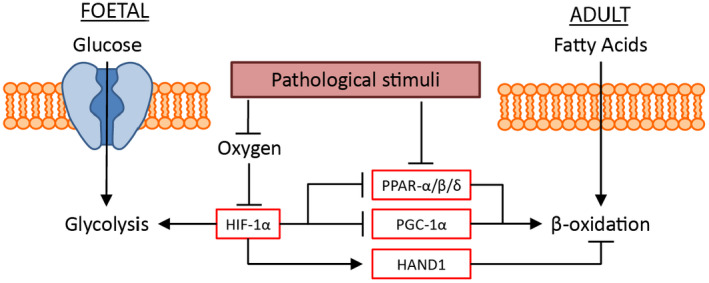
Schematic representation of the metabolic cardiac foetal reprogramming. During cardiogenesis, the cardiac tissue is primarily reliant on glycolysis for its energy requirements. This reliance on glycolysis is regulated by the relative hypoxic environment and therefore the expression of HIF‐1α, which induces the expression of glycolysis‐related genes. HIF‐1α also suppresses fatty acid oxidation (β‐oxidation) by inhibiting the expression of PPAR‐α/β/δ and PGC‐1α, and inducing the expression of HAND1, which actively suppresses β‐oxidation. Following birth, and the influx of oxygen, HIF‐1α is suppressed, leading to an increase in β‐oxidation, and a reduced utilization of glycolysis for energy production. Upon the induction of cardiac injury, there is a re‐expression of HIF‐1α leading to the inhibition of β‐oxidation and an increased reliance on glycolysis.

The transition from glycolysis to fatty acid oxidation is brought about by the shift from a relatively hypoxic environment of the foetus to physiological normoxia attained shortly after birth (Fig. 1) [25]. Subsequently, normalized oxygen tension allows for prolyl hydroxylase‐mediated degradation of HIF‐1α, leading to abrogated expression of the aforementioned HIF‐1α target genes. Amongst these target genes is *HAND1*, a transcription factor that inhibits lipid oxidation, leading to repression of mitochondrial energy generation [26]. Additionally, HIF‐1α hampers lipid oxidation through inhibition of the peroxisome proliferator‐activated receptor alpha (PPARα)/retinoid X receptor (RXR) heterodimer [27]. The transitions into a more mature heart coincides with a dramatic increase in the expression levels of PPAR‐α and PPAR‐β/δ, which are the key regulators of fatty acid metabolism [28, 29, 30].

### From fatty acid oxidation (adult) to glycolysis (disease)

In the event of various pathophysiological conditions, genes that have been active during foetal development are re‐expressed (Fig. 1) [31]. Protein levels of glycolysis genes (i.e. GLUTs, PDKs and HK‐1) are lower in healthy mature hearts than in foetal hearts. Conversely, the expression of these genes is increased in failing mature hearts to levels resembling those of the foetal heart [32]. With the re‐emergence of glycolysis as the main ATP‐generating metabolic pathway, fatty acid oxidation rates are greatly reduced (the Randle cycle) [33].

During HF, cardiac metabolism reverts to a foetal pattern in which glycolysis primarily contributes to ATP production as opposed to fatty acid oxidation [34, 35]. It has been shown that glycolysis increases as fatty acid oxidation decreases during pathological hypertrophy [36, 37]. Activity levels of mediating protein change or protein expression is altered in concert with the changes of each metabolic pathway [38, 39, 40, 41]. During HF, PPAR‐α and PGC‐1α levels decrease, consequently reducing fatty acid oxidation [42]. It is hypothesized that this reduction in PPAR‐α and PGC‐1α levels is due to rising levels of HIF‐1α. Most forms of HF result in cardiac hypoxia, for example hypertrophic cardiomyocytes increase in size and consequently oxygen tension per cell decreases. Moreover, HIF‐1α was found to be increased in pressure‐overload hypertrophy [42]. As previously stated, HIF‐1α is a master switch between glycolysis and fatty acid oxidation. Once HIF‐1α levels increase in the adult heart, expression of 6‐phosphofructo‐2‐kinase (PFK2) increases, resulting in increased levels of fructose‐2,6‐biphosphate, thereby activating PFK1 and ultimately glycolysis [43].

### Clinical relevance of targeting metabolic foetal reprogramming in Heart Failure

Cardiac damage leading to HF results in a switch in energy substrate, from fatty acids (postnatal) to carbohydrates (foetal). Several studies have focused on reversing this process of foetal reprogramming, by pharmacological activation of PPAR‐α with the agonist fenofibrate. Fenofibrate was found to prevent the HF‐induced reduction in β‐oxidation with improved cardiac function during the progression of the disease, in dogs with pacing‐induced HF [44]. However, it was unable to prevent end stage heart failure, since eventually filling pressures were elevated in both groups. Interestingly, in pigs with pacing‐induced HF, fenofibrate was found to increase the expression of PPAR‐α‐activated genes, prevent LV hypertrophy, and delayed the development of LV dilation and dysfunction [45]. However, in this study, β‐oxidation was not assessed. Conversely, various studies have also focused on inhibiting β‐oxidation in the failing heart, by pharmacologically targeting carnitine palmitoyltransferase type 1 (CPT‐1), the major source for fatty acid uptake in the mitochondria. In the experimental setting, inhibition of CPT‐1 was found to result in an overall improvement of cardiac function [46, 47]. Following these promising results, several trials were preformed to assess the potential beneficial effects of inhibiting CPT‐1 in the clinical setting [48, 49, 50, 51, 52]. The CPT‐1 inhibitor etomoxir was initially tested in a nonplacebo‐controlled pilot study enrolling 10 patients suffering from congestive HF and was found to improve clinical status, central haemodynamics at rest and during exercise, and left ventricular ejection fraction [48]. The CPT‐1 inhibitor perhexiline was also found to improve overall patient outcome. In a double‐blinded study, 56 chronic HF patients were randomized into either perhexiline or placebo group [51]. Perhexiline was found to improve ventricular ejection fraction, symptoms, and resting and peak stress myocardial function. In an additional study, 46 patients with nonobstructive hypertrophic cardiomyopathy, perhexiline was found to ameliorate cardiac energetic impairment, correct diastolic dysfunction, and increase exercise capacity [52]. Overall, these trials did demonstrate an improvement in patient outcome, however larger clinical trials will have to be conducted in HF patients to truly assess the potency of CPT‐1 inhibition. Based on these studies, it is still uncertain whether the switch in energy substrates resulting from cardiac injury is an adaptive or maladaptive response, and further research should be done to resolve this. However, it does demonstrate the potential of therapeutic intervention targeting foetal reprogramming holds.

## Foetal reprogramming in cardiac contractile machinery

The re‐emergence of foetal gene expression in the heart is not only limited to a switch in energy substrate. Maturation from a foetal to an adult heart involves a steady shift from compliant (foetal) to stiffer (adult) contractile proteins. As a result of cardiac disease, the adult heart undergoes a reversion to a more compliant foetal contractile machinery. This turnover has been highly studied in the sarcomere, which gives cardiac muscles their striated appearance and is responsible for the contractile function. The most abundant sarcomeric proteins are myofilament proteins (myosin and actin), regulatory proteins (troponins and tropomyosin) and cytoskeletal proteins (myosin binding protein C and titin). Several isoforms exist of each sarcomeric protein and it is the level of expression of these isoforms that determine the function of the cardiac sarcomere. The turnover of the sarcomeric proteins during cardiac development and disease has been extensively studied in rodent models, and to a lesser extent in the human setting (Table 1). The sarcomeric protein composition and distribution in rodent models is somewhat different from that of the human setting, and it is therefore not always possible to extrapolate the rodent findings to the clinical setting.

**Table 1:** Expression of sarcomeric proteins in foetal, adult and diseased hearts

	Foetal	Adult	Disease
Myosin heavy chain^a^			
α‐MHC	↓	↑	↓
β‐MHC	↑	↓	↑
Myosin light chain			
MLC‐1	↑	↓	↑
MLC‐2	↓	↑	↓
Actin^b^			
α‐Skeletal actin	↑	↓	↑
α‐Cardiac actin	↓	↑	↓
Troponin			
TnT_foetal_	↑	↓	↑
TnT_adult_	↓	↑	↓
TnI_foetal_	↑	↓	↑
TnI_adult_	↓	↑	↓
Titin			
N2BA	↑	↓	↑
N2B	↓	↑	↓

^a^ The ratio of α/β‐MHC is different in humans.

^b^ It is unknown if this switch occurs in the human setting.

### Myosin

Myosin heavy chain (MHC) is the so‐called ‘molecular motor’ protein of the sarcomere, which together with actin is responsible for the contraction of the cardiomyocyte, consuming ATP as the energy source to produce tension. Within cardiomyocytes there are two main isoforms of MHC, the slow twitch, β‐MHC, and the fast α‐MHC. α‐MHC has a higher ATPase activity and shortening velocity, compared to β‐MHC, therefore, hearts expressing α‐MHC possess more rapid contractile velocity than hearts expressing β‐MHC. Besides the MHC isoforms, the motor function of myosin is also regulated by the myosin light chain (MLC). Similar to MHC, the human heart expresses two isoforms of MLC, the essential (MLC‐1) and regulatory (MLC‐2). MLC‐1 has been suggested to act as a MHC/actin tether, whilst MLC‐2 slows the rate of tension development of myosin [53, 54].

The turnover in MHC in the rodent heart has been extensively studied. During cardiac development, the rodent heart switches from MHC‐β to MHC‐α, and upon cardiac injury the heart reverts back to the expression of MHC‐β. On the other hand, in human cardiac development, both α‐MHC and β‐MHC are expressed, and as the heart matures β‐MHC becomes the predominant isoform. As a result of cardiac damage, the expression levels of both isoforms is reduced and reverts back to a foetal‐like expression pattern [36, 54, 55, 56, 57, 58].

Similar to the MHC, the human heart expresses both MLC‐1 and MLC‐2 isoforms. During development, MLC‐1 is primarily expressed in the whole heart. After birth MLC‐1 expression declines rapidly and is replaced by MLC‐2 in the ventricles. However, in response to hypertrophy, ischaemia or dilated cardiomyopathies, MLC‐1 is re‐expressed [54]. Recently, it has been observed that this switch to MLC‐1 expression results in a structural change enabling cardiomyocytes to adjust to enhanced work load, by improving power output and cardiac contractility [53, 54].

These findings suggest that the re‐expression of the foetal‐like myosin, both MHC and MLC, isoforms can be considered a molecular adaptation mechanism to compensate for an increased work demand or impaired sarcomeric function.

### Actin

In mammals, actins are encoded by a multigene family, of which two main sarcomeric actin isoforms exist in cardiomyocytes: α‐skeletal and α‐cardiac actin. During cardiac development, α‐skeletal actin is primarily expressed in the foetal and neonatal hearts and as the heart matures α‐skeletal actin is slowly replaced by α‐cardiac actin [57, 59, 60, 61, 62]. Exposure to pressure‐overload hypertrophy in rats was found to result in a turnover from α‐cardiac actin to α‐skeletal actin expression [57, 61, 62, 63]. Similarly, in cultured neonatal cardiomyocytes exposed to α1‐adrenergic agonists or growth factors TGFβ1 and bFGF, α‐skeletal actin mRNA was observed to be significantly increased [64]. Several studies have examined if in humans an actin isoform switch takes place during development and disease; however, the results have been contradictory and as such it still remains unclear if in humans this switch takes place [57, 58, 65, 66, 67]. Noteworthy is the observation that α‐skeletal actin, when compared to α‐cardiac actin, can strongly promote the contractility of the myocardium by activating α‐MHC’s ATPase activity to a larger extend [68]. This suggests the turnover from α‐cardiac to α‐skeletal actin to be an adaptive response to maintain cardiac contractility due to the increased presence of α‐MHC in the myocardium.

### Troponin

Troponin is part of the myofibrillar contractile complex, involved in controlling muscular contraction by regulating the myofibrillar responsiveness to calcium and adrenergic stimulation. Troponin consists of 3 subunits, troponin C, T and I (TnC, TnT and TnI, respectively). During normal cardiac development in rats, both TnT and TnI switch from their respective foetal to the adult isovorms [67, 69, 70]. Rat hearts when exposed to cardiac injury demonstrate a re‐expression of the foetal isoforms of TnT and TnI [67, 70, 71]. Several studies have shown that the foetal TnT and TnI isoforms have a lower calcium sensitivity, adrenergic sensitivity, ATPase activity and cardiac muscle relaxation [67, 70]. Suggesting that the re‐expression of the foetal TnT and TnI is either a mechanism of pathologic change or a maladaptive process induced by the pathological changes of HF.

### Titin

Titin is a large protein that has been characterized as molecular spring, with its elastic properties defining the passive mechanical properties of cardiomyocytes. In humans, titin is encoded by a single gene (*TTN*) containing 363 exons that are differentially spliced, creating the stiffer N2B (short molecular spring) and the more compliant N2BA (long molecular spring) isoforms [67, 72, 73]. Both isoforms are co‐expressed in the sarcomere, and the degree of expression of each isoform adjusts the passive stiffness [67, 72, 73]. When looking at neonatal pig hearts, it was observed that these had a higher abundance of the complaint N2BA titin isoform, whilst adult pig hearts demonstrated a shift towards the stiffer N2B isoform [67, 72]. This observation suggests that as the heart matures there is an increase in passive myocardial stiffness, which could play a role in adjusting for diastolic function during development. Upon cardiac damage, a shift from the N2B to N2BA has been observed, in both the experimental and clinical setting [67, 72, 73, 74]. This shift causes a reduction in titin‐derived myofibrillar stiffness, which can lead to a decrease in cardiac output.

### Clinical relevance of targeting contractile foetal reprogramming in heart failure

Several studies have aimed at understanding the therapeutic potential of targeting the foetal reprogramming of the cardiac contractile machinery. These studies have primarily focused on improving the contractility of the cardiomyocytes in the failing heart by targeting myosin. Similar to the foetal human heart, the diseased adult myocardium predominantly expresses more β‐MHC, an MHC isoform characterized for lower ATPase activity and reduced shortening velocity when compared to α‐MHC. As a consequence, the diseased myocardium has a reduced contractile capacity. Omecamtiv mecarbil (OM) is a selective, small‐molecule cardiac myosin activator that binds to the catalytic domain of myosin, thereby increasing cardiac contractility without affecting cardiomyocyte calcium concentrations or myocardial oxygen consumption [75]. OM has been shown in improve cardiac muscle function in animal models for HF [76, 77, 78, 79]. Additionally, OM was found to have properties protecting the heart against ischaemia and reperfusion injury [80]. Whilst OM does not directly reverse cardiac foetal reprogramming of the myosin, it enables the present β‐MHC to have an improved contractile capacity, similar to that of α‐MHC [77, 81]. Following these positive results in the experimental setting, several clinical trials have been performed with the administration of OM to HF patients. In the ATOMIC‐AHF study evaluated the pharmacokinetics, pharmacodynamics, tolerability, safety and efficacy of intravenous OM administration in patients with acute HF [82]. In the 606 patients included, OM administration did not meet the primary end‐point of dyspnoea improvement; however, it was well tolerated, increased systolic ejection time, and it may have improved dyspnoea in the high‐dose group [82]. In the COSMIC‐HF study, a randomized, double‐blinded, placebo‐controlled study, the pharmacokinetics of OM administration were assessed in HF patients, and changes in cardiac function and ventricular diameters [83]. A total of 299 patients were enrolled, 150 received OM whilst 149 were placed in the placebo group. OM dosing achieved plasma concentrations associated with improved cardiac function and decreased ventricular diameter [83]. Finally, the ongoing GALACTIC‐HF trial, a randomized, double‐blinded, placebo‐controlled, event‐driven cardiovascular outcome trial, is assessing the potential of OM treatment for its ability to improve symptoms, prevent clinical HF events and delay cardiovascular death in patients with chronic HF [84]. More than 8,000 chronic HF patients have been enrolled and randomized to either oral placebo or OM treatment. The GALACTIC‐HF trial is the first trial examining whether selectively increasing cardiac contractility in patients with HF with reduced ejection fraction will result in improved clinical outcomes [84].

## Foetal reprogramming in cardiac electrophysiology

Besides the switches in metabolism and contractile machinery, the reversion to a more foetal‐like state in response to cardiac injury has also been observed in the mechanisms regulating the electrophysiology of cardiomyocytes. Cardiac electrophysiology is in large part governed by the expression of ion channels, gap junctions and the calcium homeostasis.

### Ion channels

**Fig. 2:**
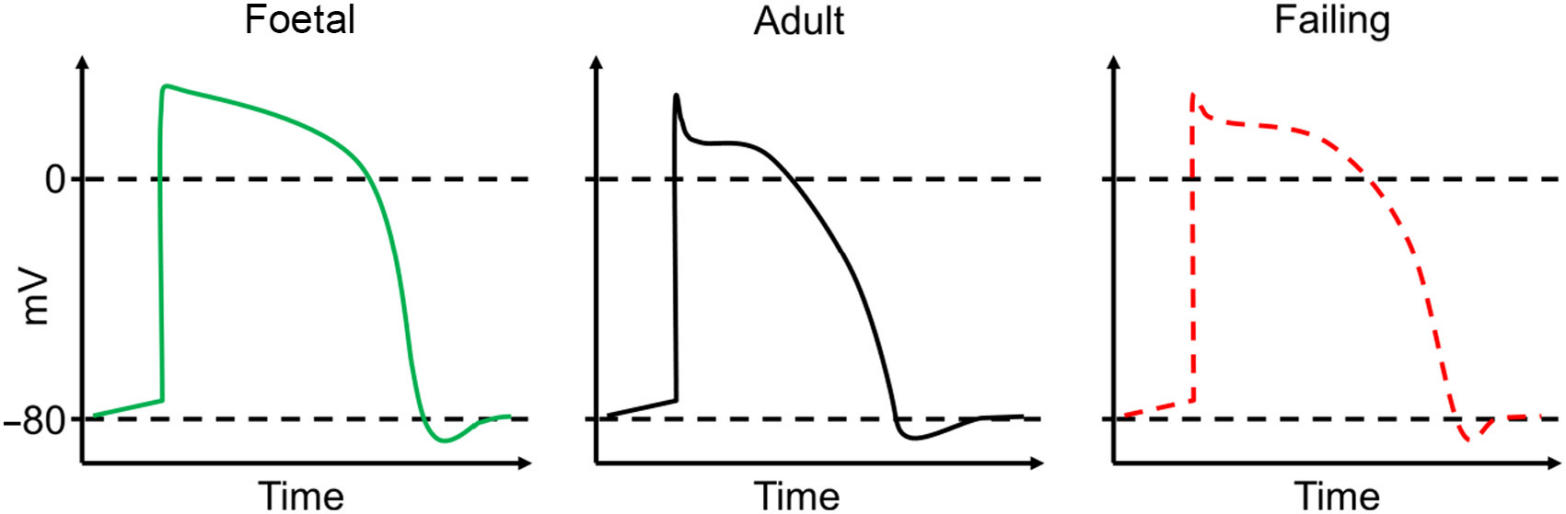
Schematic representations of the foetal, adult and diseased action potential. (LEFT) In the foetal stages of cardiac development, the heart has a prolonged action potential primarily due to a reduced expression of potassium channels. (MIDDLE) A schematic representation of an adult heart action potential. Compared to the foetal heart, the adult heart has an increased expression of potassium channels, sodium channels, and a reduction in calcium channels. (RIGHT) Following cardiac injury, the myocardium has a reduced expression of potassium channels and sodium channels coupled to an increase in the expression of calcium‐sensitive channels. This switch leads to an increase in action potential, reminiscent of the foetal action potential.

Ion channels are essential for the generation and propagation of the current that enables the heart to perform its function. Extensive research has been done to understand the electrophysiological changes that occur during cardiac maturation. It has been observed that the excitability, action potential properties, contractility and relaxation of the foetal and adult heart differ significantly from each other [85]. As a result, the foetal heart expresses different genes involved in the generation and propagation of the action potential then the adult heart. In mice during cardiac development from foetal to adult, there is an up‐regulation of genes involved in the I_k1_ (*KCNH2*), I_to_ (*KCND2* and *KCND3*), I_kr_ (*KCNH2*), I_ks_ (*KCNQ1*), I_Ca,L_ (*CACNA1C*), I_Na_ (*SCN5A*), I_f_ (*HCN1* and *HCN4*) currents, and a down‐regulation of genes involved in the I_Ca,T_ (*CACNA1H*) and NCX (*NCX1*) currents [85, 86, 87, 88, 89]. The reduced expression of potassium channels in the foetal heart coincides with the observation that these hearts have a less negative resting and longer action potentials compared to adult hearts [85]. The observed lower expression of I_Ca,L_ and higher I_Ca,T_ and NCX is consistent with the greater importance of alternate calcium‐entry pathways in the foetal versus adult hearts [85]. The increase in sodium channel expression in adult mouse hearts may be necessary for rapid activation of the much larger heart [85]. Similarly, studies comparing human cardiomyocytes derived from human induced pluripotent stem cells (hiPSC) or from human embryonic stem cells (hESC), to adult tissue have shown similar findings [90, 91]. Interestingly, in cardiac tissue of patients diagnosed with end stage HF, the major sodium (I_Na_), potassium (I_to_, I_K1_, I_Kr_ and I_Ks_) and calcium (I_ca,L_) ion channels are significantly repressed, whilst I_ca,T_, NCX and I_f_ (*HCN4*) are up‐regulated [89, 92, 93]. Therefore, the heart responds to an increased load by decreasing the potassium currents, thereby prolonging the action potential and increasing the calcium within the cardiomyocytes, leading to increased contractility (Fig. 2). These findings suggest that as a result of cardiac injury, the heart undergoes ion channel remodelling, resulting in an expression profile similar to that of the foetal heart. Initially, these changes are adaptive, however in the long run they can become maladaptive by increasing the changes of arrhythmias [94, 95].

### Gap junctions

Gap junctions are clusters of intercellular channels, assembled forming connexins. These connexins function as pathways enabling electrical current propagation, which controls the rhythm of the heart. As is the case in most tissues and organs, multiple connexins are expressed in the heart, specifically connexin43, connexin 40 and connexin45 [96, 97]. The presence of each connexin type varies in relative to quantities depending on the functional specialization of each subset of cardiomyocytes. The most predominant gap junction protein in the adult heart is connexin43, expressed highly in all cardiomyocytes subsets of the heart [96, 97]. In the sinoatrial node, the site of impulse generation, and the atrioventricular node, the site where impulse is slowed before being routed to the ventricles, cardiomyocyte gap junctions are formed by connexin43 and connexin45, associated with slow conductance [97]. Cardiomyocytes of the His–Purkinje conduction systems are mainly characterized by the expression of connexin40, a connexin associated with high conductance, which facilitates rapid distribution of the impulse throughout the working ventricular myocardium [97].

It has been well established, in rodents and the human setting, that as the heart matures the expression of connexin43, connexin 40 and connexin45 are progressively increased [96, 97, 98, 99]. This increase in the density of gap junctions in the developing heart is directly linked to an increase in conduction velocity. It has been shown that upon cardiac injury, in both rodent HF models and in the human clinical setting, connexin43 expression is not only drastically reduced (±50%), but the remaining connexin43 gap junctions are also highly disorganized [97, 100, 101]. This decrease in connexin43 expression is also associated with an increase in connexin40 expression [97, 100, 101]. Whether this increase in connexin40 expression is a result of reduced connexin43 levels, or whether it is an adaptive response leading to increased impulse propagation throughout the myocardium is unknown [97, 100, 101]. It has been suggested that the reduction in cell‐to‐cell coupling in HF results in an increase in the QT‐interval, action potential prolongation and increased risk of arrythmias [97, 100, 101].

### Calcium homeostasis

Intracellular calcium homeostasis (release and uptake) plays an essential role in regulating excitation–contraction coupling and in modulating systolic and diastolic function in the heart. Calcium ions are initially imported into the cell through the plasma membrane by means of the I_Ca,L_ current, which is generated as a result of the depolarization of the plasma membrane. Calcium entry from the plasma membrane activate the ryanodine receptors (RyR), which results in an efflux of calcium ions from the sarcoplasmic reticulum (SR), in a process known as calcium‐induced calcium release. The released cytoplasmic calcium interacts with calcium‐sensitive proteins (i.e. TnC) controlling the force and rate of contraction. The cytoplasmic calcium is then pumped back into the SR, by SR calcium‐ATPases (SERCA) activity, and out through the plasma membrane, by NCX activity. The expression of the I_Ca,L_ current, TnC and NCX in cardiac development and disease has been covered in the previous section; here, we will focus on the expression of RyR and SERCA during cardiac development and disease.

Calcium homeostasis has been extensively studies in both cardiac development and cardiac disease, especially in terms of the expression of RyR and SERCA. There are three major isoforms of the RyR, of which RyR2 is the major SR calcium‐release channel involved in excitation–contraction coupling in the heart. SERCA functions by transferring calcium from the cytosol to the SR at the expense of ATP during muscle contraction, in the cardiomyocytes the major SERCA isoform is SERCA2, encoded by *ATP2A2*. In studies exploring cardiac development in rodents and in hESC/hIPCS cardiac differentiation, it has been observed that the expression of both RyR2 and SERCA2 is up‐regulated [85, 102, 103, 104]. The greater expression of the calcium handling proteins (*ATP2A2*, *RyR2* and *CACNA1C*) during cardiac development may be essential for the stronger mechanical function required to provide the blood supply to much larger adult bodies [85, 102, 103, 104].

The expression of RyR2 in HF has been controversial, several studies have shown a reduction in the expression levels, back to foetal levels, in rodent and human HF [105, 106], however numerous studies have also shown no change in the expression of RyR2 in HF [105, 106]. Therefore, the exact expression and involvement of RyR2 during HF remains uncertain. Interestingly, studies have demonstrated that during HF, RyR2 are hyperphosphorylated resulting in a leaky RyR2 channel and reduced SR calcium content [105, 107]. On the other hand, SERCA2 expression, which is increased during cardiac developing, has been shown to be substantially reduced in HF, in both rodent and human models [85, 108, 109]. Furthermore, a decrease in phospholamban (PLN), a regulator of SERCA2 activity, phosphorylation in HF has been described, which further depressing the function of SERCA2 [107]. Combined, the reduction of SERCA2 expression/activity and SR leakage by hyperphosphorylated RyR2 channels lead to reduced SR calcium content, resulting in reduced SR calcium release, myofilament activation and contractility [107].

### Clinical relevance of targeting electrical physiology foetal reprogramming in Heart Failure

To help extrapolate the observations demonstrating the process of foetal reprogramming on the electrical physiological level in HF, a number of studies have explored modulating ion channels and calcium handling in the failing heart. Such studies have demonstrated that by overexpressing SERCA2, thus reverting cardiac foetal reprogramming, in transgenic rodent models for HF, these animals have improved cardiac function and are less prone to develop HF following myocardial injury [110, 111, 112]. Similarly, overexpression of SERCA2 by means of adenoviral gene transfer, in HF models, has also demonstrated beneficial effects [113, 114]. Following these positive results observed from modulating the cardiac foetal reprogramming of SERCA2 in the experimental setting, clinical trials with SERCA2 gene therapy have been performed. The initial findings were encouraging, demonstrating improved cardiac function, decreased HF symptoms and reduced mortality in patients with advanced HF [115, 116]. However, a recent study utilizing the same SERCA2 gene therapy showed no improvement on ventricular remodelling in patients with advanced systolic HF [117]. In contrast to boosting gene expression, the application of antisense oligonucleotide therapy is becoming more feasible (technically and financially) to intervene in pathways at different levels [118, 119]. Consequently, SERCA2 function has been successfully enhanced by antisense oligonucleotide therapy in mice targeting PLN, a regulator of SERCA2 activity. Besides utilizing gene therapy as a therapeutic strategy, pharmacological agents that restore SERCA2 function have also been explored. One such agent is istaroxime, which functions by stimulating SERCA2 activity and indirectly inhibiting NCX function by increasing intracellular sodium levels [120]. Treatment with istaroxime in animal models of HF have shown improved cardiac function with no adverse effects [121, 122]. Following these animal studies, a clinical trial evaluating the effects of acute istaroxime administration in HF patients has demonstrated an improvement in cardiac function in these patients [123, 124, 125]. Although such a pharmacological intervention does not directly target cardiac foetal reprogramming, like direct gene therapy does, it does ensure that the remaining SERCA2 is more active, therefore compensating for the lack of SERCA2 expression. Taken together, these studies have further demonstrated the potential of targeting foetal reprogramming in cardiac electrical physiology in HF.

## Cardiac neurohormonal foetal reprogramming

Cardiac foetal reprogramming is not only limited to the metabolism, contractile machinery and electrophysiology, but also occurs in the expression of cardiac neurohormones. Specifically, foetal reprogramming has been observed in the expression of atrial and brain natriuretic peptides.

The atrial natriuretic peptide (ANP) was the first natriuretic peptide identified in 1981 [126]. Since then extensive research has been done on ANP, whose primary function has been identified to reduce plasma volume, and therefore blood pressure, by increased renal excretion of salt and water, vasodilation, increased vascular permeability [127]. In the adult murine and human heart, the atrium is the major source of ANP expression; however, during cardiac development, ANP expression is primarily localized to the ventricles [128]. As the heart matures, the expression of ANP in the ventricles is significantly reduced in the ventricles [128]. The primary stimuli for ANP expression is stretch, thus as a result of cardiac hypertrophy, remodelling and HF, ANP is significantly expressed in the ventricles returning to foetal expression levels [128, 129].

Following the discovery of ANP, a second natriuretic peptide was identified in the brain, brain natriuretic peptide (BNP) [128]. Although initially isolated and characterized in the brain, BNP was later identified as being predominantly expressed in the heart ventricles [128]. Furthermore, BNP has a similar mode of action as ANP, that is to lower blood volume, reduce cardiac output and systemic blood pressure. Additionally, BNP mimics the expression profile of ANP during cardiac development, with BNP levels being significantly reduced in the adult compared to the foetal myocardium [128, 129]. Upon myocardial stretch, BNP, like ANP, is re‐expressed by the ventricles [128, 129].

In recent years, it has been well established that the re‐expression of both ANP and BNP has a cardioprotective effect in the failing myocardium [130]. In the human setting, the actions of BNP are of particular interest. BNP not only helps unload the failing heart, by reducing preload, facilitate renal excretion of salt and water, and to inhibit the renin–angiotensin system, but it is also involved in the inhibition of the sympathetic drive to the heart, enhancement of the parasympathetic cardiac reflex, and inhibition of pathological cardiac hypertrophy [130]. Thus, the re‐expression of ANP and BNP in the failing heart is an adaptive response that helps to protect the failing heart.

### Clinical relevance of targeting neurohormonal foetal reprogramming in Heart Failure

The notion that neurohormonal foetal reprogramming occurs in the failing heart has been widely adapted in the form of biomarkers, where ANP and BNP have been characterized to associate with HF disease severity and progression [131]. However, it has recently also become evident that neurohormonal foetal reprogramming can also serve as an ideal handle for intervention, specifically BNP. As previously mentioned, the re‐expression of BNP results in several cardioprotective effects. Based on these, two main therapeutic strategies have been employed to further increase the levels of natriuretic peptides in HF patients. The first approach has been to target neprilysin, the enzyme responsible for the degradation of natriuretic peptides. A prodrug that strongly inhibits the activity of neprilysin, sacubitril, has been shown to not only have beneficial effects in models for HF, but also in the clinical setting [132, 133, 134, 135]. The combination of valsartan/sacubitril has now become a class I recommendation in the treatment of heart failure with a reduced ejection fraction [136]. The second approach has been to administer engineered recombinant natriuretic peptides, which mimic the effects of the endogenous natriuretic peptides. These recombinant natriuretic peptides have also demonstrated beneficial effects in both HF animal models and in the clinical setting [137, 138, 139].

## Exploring unknown elements of the foetal programme to uncover new therapeutic avenues

The heart exposed to stress undergoes physiological changes bringing it back to a more foetal‐like state, in other words cardiac foetal reprogramming. Cardiac damage leading to HF results in a switch in energy substrate, from fatty acids (postnatal) to carbohydrates (foetal). Similarly, the contractile machinery of the heart reverts back to a more compliant state, as observed in the foetal heart. Finally, cardiac electrophysiology, governed by ion channels, gap junctions and calcium homeostasis, also switches to a state similar to that observed in the foetal heart. Initially, the process of cardiac foetal reprogramming seems to be an adaptive response to cope with adverse remodelling in the heart, and as such a consequence of cardiac injury. However, as time progresses, these changes are detrimental to the myocardium and add further insult leading to disease progression. Therefore, targeting cardiac foetal reprogramming could be ideal for therapeutic interventions.

**Fig. 3:**
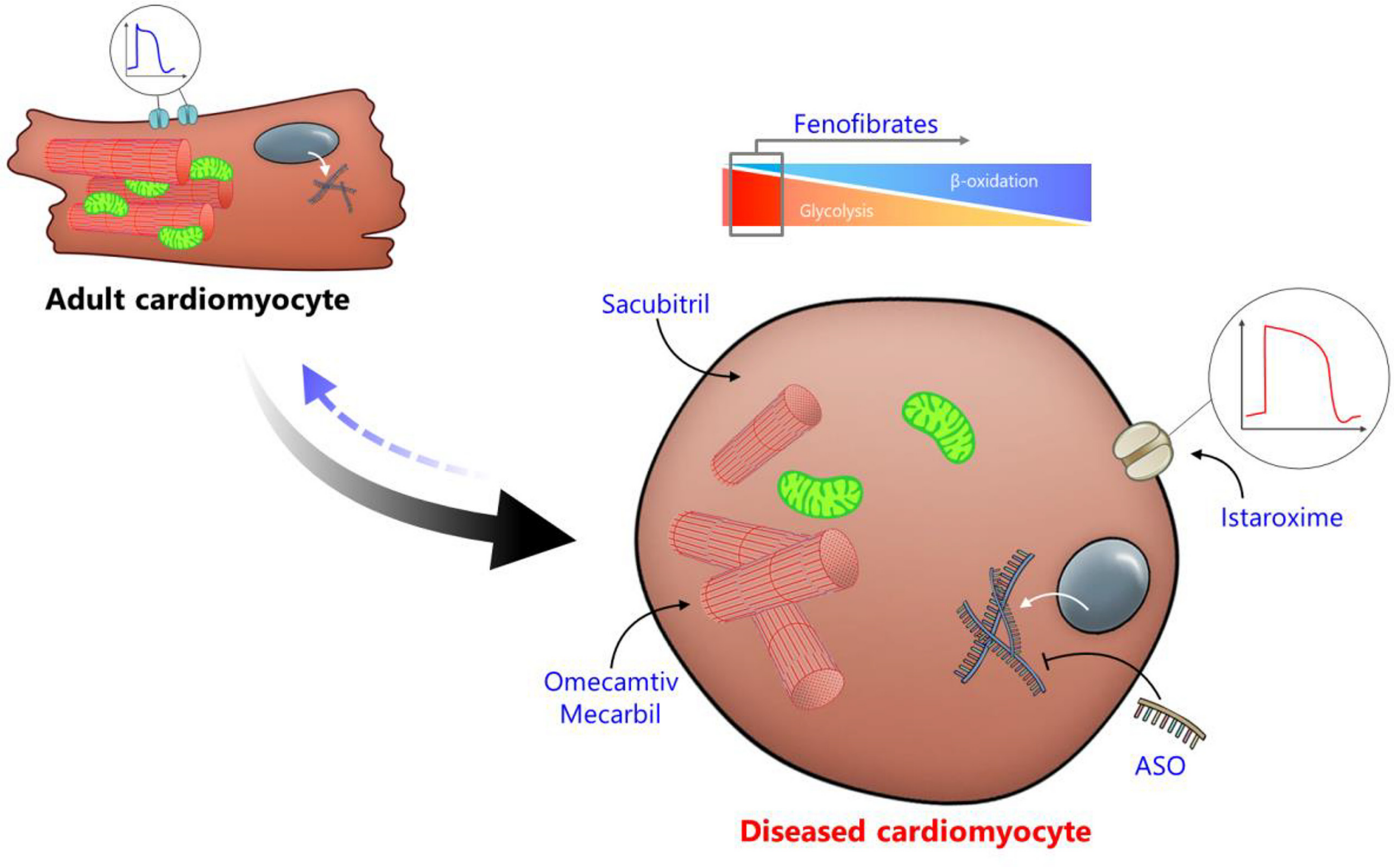
Therapeutic strategies to revert foetal reprogramming in disease. Regained foetal characteristics in diseased adult cardiomyocytes are potential therapeutic targets based on specific dysfunctional aspects. Therapeutic strategies marked in blue may improve cardiomyocyte function and may, therefore, revert pathological foetal reprogramming (blue arrow). Fenofibrate, a PPAR‐α agonist, can prevent HF‐induced switch from β‐oxidation to glycolysis. Omecamtiv Mecarbil, a small‐molecule cardiac myosin activator, improves cardiac contractility, thereby reversing foetal reprogramming. A reduction of SERCA2 expression is a hallmark of cardiac electrical physiological foetal reprogramming, and istaroxime has been found to reverse this by improving SERCA2 activity. Re‐expressing natriuretic peptides, a prime example of cardiac foetal reprogramming, has a positive effect on cardiac outcome, Sacubitril has been found to strongly improve the presence of natriuretic peptides by inhibiting the activity of neprilysin. Antisense oligonucleotides (ASO) have demonstrated their potential to intervene in cardiac foetal reprogramming by improving the activity of SERCA2, however this approach could be extrapolated to target various other aspects of foetal reprogramming.

In the previous sections, we have demonstrate that targeting various aspects of the cardiac foetal gene programme holds great promise in improving patients’ outcome. Therefore, it is our opinion that attaining a better understanding of this process may eventually lead to better therapeutic options for HF patients. To obtain a better understanding of cardiac foetal reprogramming, studies in the field of cardiovascular research should focus on further elucidating this process. There are several ways one could go about characterizing the cardiac foetal gene programme, especially with the current improvements in the ‘omics’ techniques (i.e. genomics, proteomics and metabolomics). These techniques could lead to a better understanding of how diseased myocardium mimics the developing heart. Furthermore, pathophysiological pathways implicated in cardiac foetal reprogramming could be uncovered, leading to novel therapeutic targets. Recently, we focused on characterizing the murine cardiac foetal gene programme, by means of RNA sequencing, this has led to the identification of several new cardiac foetal genes, including genes associated with oxidative stress, extra cellular matrix composition, metabolism and signal transduction (Table 2) [140]. Of particular interest was OPLAH, a gene that encodes for 5‐oxoprolinase, an antioxidant enzyme involved in the γ‐glutamyl cycle, where it is responsible for the conversion of 5‐oxoproline, a degradation product of glutathione, into glutamate [141, 142]. OPLAH was found to be expressed during cardiac development and repressed in cardiac disease, in both the experimental and clinical settings [140, 143]. By overexpressing OPLAH, and reversing cardiac foetal reprogramming of the gene, mice were found to have improved cardiac function following myocardial infarction [140]. The improvement in cardiac function was found to result from a reduction in oxidative stress, due to a decrease in 5‐oxoproline, a strong mediator of oxidative stress [140, 144, 145]. These findings suggest OPLAH may be an interesting candidate for therapeutic intervention. Identifying drugs and or small molecules capable of enhancing OPLAH expression and/or activity may therefore lead to novel therapeutic strategies for HF patients. Interestingly, 5‐oxoproline was also found to be a potential novel biomarker for HFyy^140^. Combined, this study demonstrates the potential of characterizing cardiac foetal reprogramming to help uncover novel pathophysiological pathways which may lead to new therapeutic strategies and improved patient outcome (Fig. 3).

**Table 2:** Several known and novel members of the cardiac foetal gene program recently identified

Gene	Annotation	Developmental	Diseased
Know members of the cardiac foetal gene program			
*RYR2*	Ryanodine receptor 2, cardiac	↑	**↓**
*CACNA2D1*	Calcium channel, voltage‐dependent, alpha2/delta subunit 1	↑	**↓**
*ATP2A2*	ATPase, Ca++ transporting, cardiac muscle, slow twitch 2	↑	**↓**
Novel members of the cardiac foetal gene program			
*OPLAH*	5‐Oxoprolinase (ATP‐hydrolysing)	**↑**	**↓**
*ANXA11*	Annexin A11	**↑**	**↓**
*HADH*	Hydroxyacyl‐Coenzyme A dehydrogenase	**↑**	**↓**
*CD300LG*	CD300 antigen like family member G	**↑**	**↓**
*MCCC1*	Methylcrotonyl‐Coenzyme A carboxylase 1 (alpha)	**↑**	**↓**
*LGALS4*	Lectin, galactose binding, soluble 4	**↑**	**↓**
*CYYR1*	Cysteine and tyrosine‐rich protein 1	**↑**	**↓**
*SLCO2B1*	Solute carrier organic anion transporter family, member 2b1	**↑**	**↓**
*RALGAPA2*	Ral GTPase activating protein, alpha subunit 2 (catalytic)	**↑**	**↓**
*DNAJB1*	DnaJ (Hsp40) homolog, subfamily B, member 1	**↓**	**↑**
*THEM6*	Thioesterase superfamily member 6	**↑**	**↓**
*ETL4*	Enhancer trap locus 4	**↑**	**↓**
*ABHD14B*	Abhydrolase domain containing 14b	**↑**	**↓**
*VPS13C*	Vacuolar protein sorting 13C (yeast)	**↑**	**↓**
*OSTC*	Oligosaccharyltransferase complex subunit	**↓**	**↑**
*FREM1*	Fras1 related extracellular matrix protein 1	**↓**	**↑**
*DENND4C*	DENN/MADD domain containing 4C	**↑**	**↓**
*SNX6*	Sorting nexin 6	**↓**	**↑**
*HSP90AA1*	Heat shock protein 90, alpha (cytosolic), class A member 1	**↓**	**↑**
*PSMC3IP*	Proteasome (prosome, macropain) 26S subunit, ATPase 3, interacting protein	**↓**	**↑**
*DNAJA1*	DnaJ (Hsp40) homolog, subfamily A, member 1	**↓**	**↑**
*ITM2A*	Integral membrane protein 2A	**↓**	**↑**
*VPS13D*	Vacuolar protein sorting 13 D (yeast)	**↑**	**↓**
*PPRC1*	Peroxisome proliferative activated receptor, gamma, coactivator‐related 1	**↓**	**↑**
*SLC25A22*	Solute carrier family 25 (mitochondrial carrier, glutamate), member 22	**↑**	**↓**
*LSM1*	LSM1 homolog, U6 small nuclear RNA associated (S. cerevisiae)	**↓**	**↑**
*BANP*	BTG3 associated nuclear protein	**↓**	**↑**
*KIF26B*	Kinesin family member 26B	**↓**	**↑**
*GRB10*	Growth factor receptor bound protein 10	**↓**	**↑**

## Author Contribution

**Atze Van der Pol:** Visualization (equal); Writing‐original draft (lead); Writing‐review & editing (lead). **Martijn Hoes:** Visualization (supporting); Writing‐original draft (supporting); Writing‐review & editing (supporting).
